# Biodistribution and toxicity of radio-labeled few layer graphene in mice after intratracheal instillation

**DOI:** 10.1186/s12989-016-0120-1

**Published:** 2016-02-11

**Authors:** Liang Mao, Maojie Hu, Bingcai Pan, Yongchao Xie, Elijah J. Petersen

**Affiliations:** 1 State Key Laboratory of Pollution Control and Resource Reuse, School of the Environment, Nanjing University, Nanjing, 210093 P. R. China; 2 Material Measurement Laboratory, Biosystems and Biomaterials Division, National Institute of Standards and Technology, 100 Bureau Drive, Stop 8311, Gaithersburg, MD 20899-0001 USA

**Keywords:** Few layer graphene, Biodistribution, Toxicity, Mice, Intratracheal instillation, Occupational risk

## Abstract

**Background:**

The potential human health risks from graphene inhalation exposure have attracted substantial scientific interest as a result of the numerous exciting potential commercial applications of graphene. However, the long-term distribution of graphene in organisms after inhalation is unknown, largely as a result of challenges associated with accurate graphene quantification.

**Methods:**

Carbon-14 labeled FLG was used to quantify the *in vivo* distribution of FLG in mice after oral gavage or intratracheal instillation for up to 3 or 28 days after exposure, respectively.

**Results:**

Intratracheally instilled FLG was mainly retained in the lung with 47 % remaining after 4 weeks. Exposure to non-labeled FLG resulted in dose-dependent acute lung injury and pulmonary edema, but these effects were alleviated with time despite the continued presence of FLG in the lungs. One percent and 0.18 % of the intratracheally instilled FLG was present in the liver and spleen, respectively, after 14 days by passing through the air-blood barrier, a finding supported by the results of oral gavage experiments which did not show detectable absorption through the gastrointestinal tract. In addition, 46.2 % of the intratracheally instilled FLG was excreted through the feces 28 d after exposure.

**Conclusions:**

Quantitative measurements revealed the elimination mechanism for FLG and its biodistribution for two exposure pathways. Graphene persistence in the lung only caused transient pulmonary effects. The *in vivo* distribution, elimination, and toxicity results provided here measured using a robust quantitative method support the human health risk assessment of graphene.

**Electronic supplementary material:**

The online version of this article (doi:10.1186/s12989-016-0120-1) contains supplementary material, which is available to authorized users.

## Background

Graphene is a flat monolayer of carbon atoms tightly packed into a two-dimensional lattice and has a number of potential applications due to its unique intrinsic properties [1–5]. When considering the potential human health risks of nanoparticles, inhalation is thought to be the exposure route of highest concern [6–9]. The calculated deposition fraction of few layer graphene (FLG) with different lateral dimensions ranging from 0.001 to 100 μm in the nasopharyngeal, tracheobronchial, and alveolar regions revealed that there would be substantial deposition of these nanoplatelets throughout the respiratory tract [6].

Findings from several recent studies indicate that graphene and graphene oxide (GO) may induce the acute inflammation and pulmonary fibrosis in mice lungs [6–9]. However, these studies have not quantified the persistence of graphene/GO in the mice lungs after a long exposure time (Additional file 1: Table S1). Nanoparticles may translocate to extrapulmonary organs and may be redistributed to other tissues after deposition in the lung [10–12]. Thus, knowledge of graphene biodistribution in mice after inhalation remains a key research gap. One significant challenge for measuring graphene biodistribution is that methods are not available for reliable graphene quantification graphene in tissues. While a single study reported the short-term biodistribution and pulmonary toxicity of GO in mice by using ^125^I-labeled GO [13], quantitative results were only provided for the first 12 h and the biodistribution results from this study are questionable because a fraction of the labeled ^125^I ions was found to be released from the GO conjugate [14]. In addition, the structure of graphene substantially differs from that of GO, which contained five- and six-membered-ring lactols and more than 40 % oxygen, and thus the pulmonary toxicity and biodistribution of graphene may similarly differ [15, 16].

In this study, ^14^C-labeled FLG were utilized to quantify the *in vivo* distribution and excretion of graphene in mice up to 28 days and 3 days after intratracheal instillation or oral gavage, respectively. The ^14^C-labeled graphene was utilized because the carbon-14 atoms were stably bound to the graphene skeleton. Potential toxicological effects from non-labeled FLG exposure were also assessed. Both the acute and long-term toxicity after intratrachael instillation and the impact of graphene on the microbial community composition of intestinal flora after oral gavage were evaluated.

## Results and discussions

### Graphene properties

**Fig. 1:**
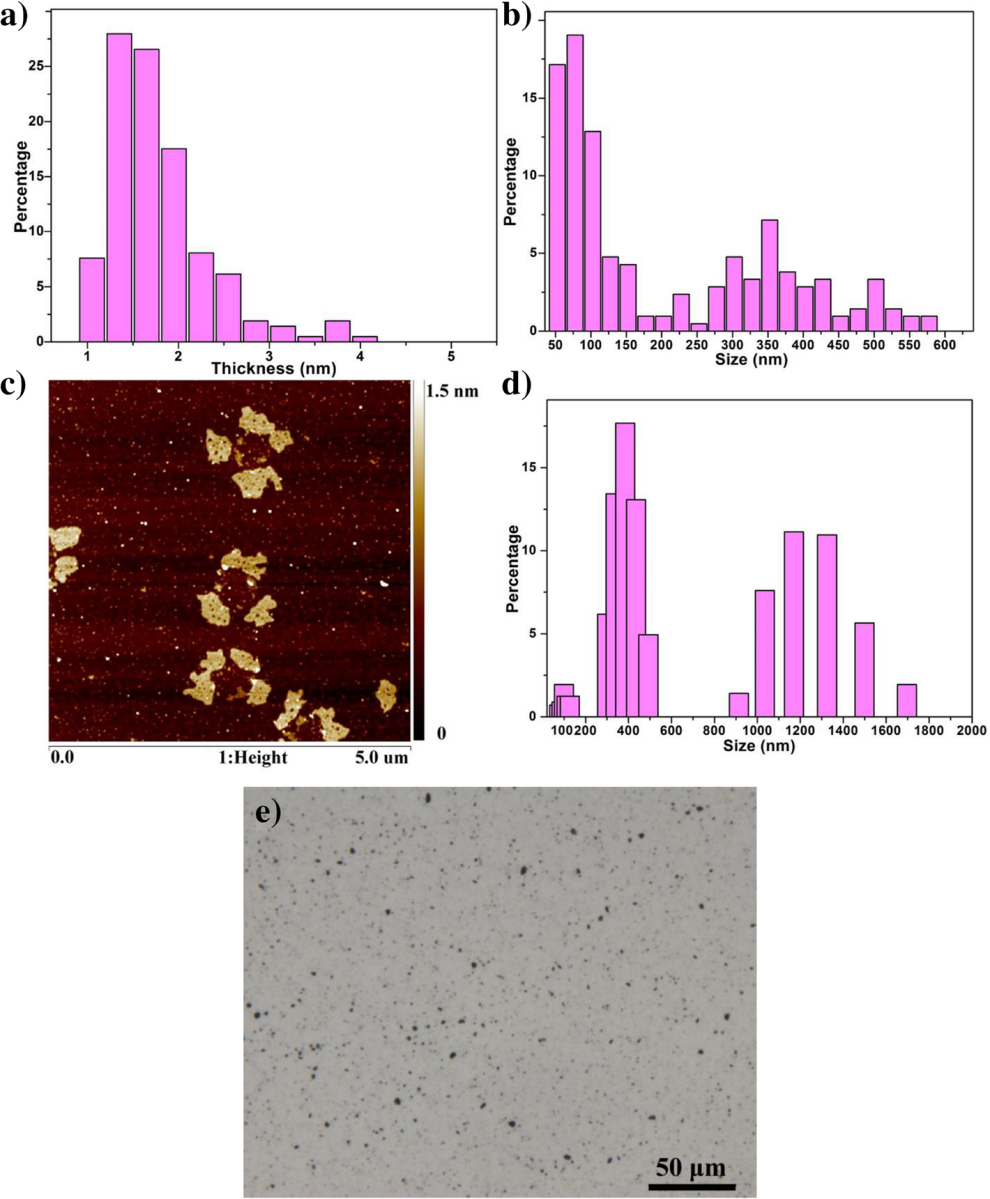
Characterization of FLG using AFM and DLS. **a** Histogram of flake thickness for FLG (*n* = 214); **b** Histogram of lateral flake size for FLG (*n* = 214); **c** Representative AFM image of FLG deposited onto mica; **d** Size distribution of FLG dispersed in 0.1 % Tween 80 saline measured by DLS; **e** FLG suspension (0.5 mg/mL, suspend in 0.1 % Tween 80 saline) observed under light microscopy

The atomic ratio of C:O in the FLG was determined using X-ray photoelectron spectroscopy to be 89:6 (the remaining 5 % is 1.4 % of H and 3.6 % of N) [17]. Notably, the oxygen was introduced by the adding ^14^C-phenol in FLG synthesis, not by oxidation. In addition, the oxygen percentage is ~6 %, which is significantly lower than that for GO [15, 16]. The specific radioactivity of the purified FLG was 16.12 ± 0.59 mCi g^-1^ (*n* = 3). From the characterization results of atomic force microscopic (AFM) (see Fig. 1), the thickness of suspended FLG was measured to be 0.97 to 3.94 nm and 72 % of total count was in range of 1.2 to 2.1 nm. Given that the graphene interlayer distance is 0.35 ± 0.01 nm [18, 19], the FLG mainly consisted of 4 to 6 layers (>72 %). In addition, AFM measurements showed that the FLG graphene had a continuous lateral size distribution from 60 to 590 nm and two main peaks at 90 nm and 365 nm. The hydrodynamic diameter of graphene suspended in 0.1 % Tween 80 saline has three peaks with average sizes at 100, 390 and 1250 nm (Fig. 1d). The smaller two peaks are in accordance with results measured by AFM, while the larger peak may be due to formation of graphene aggregates. Light microscopy images showed large black dots which were regarded to be graphene aggregates (Fig. 1e), which would agree with the dynamic light scattering (DLS) results.

### Distribution of graphene in mice

**Fig. 2:**
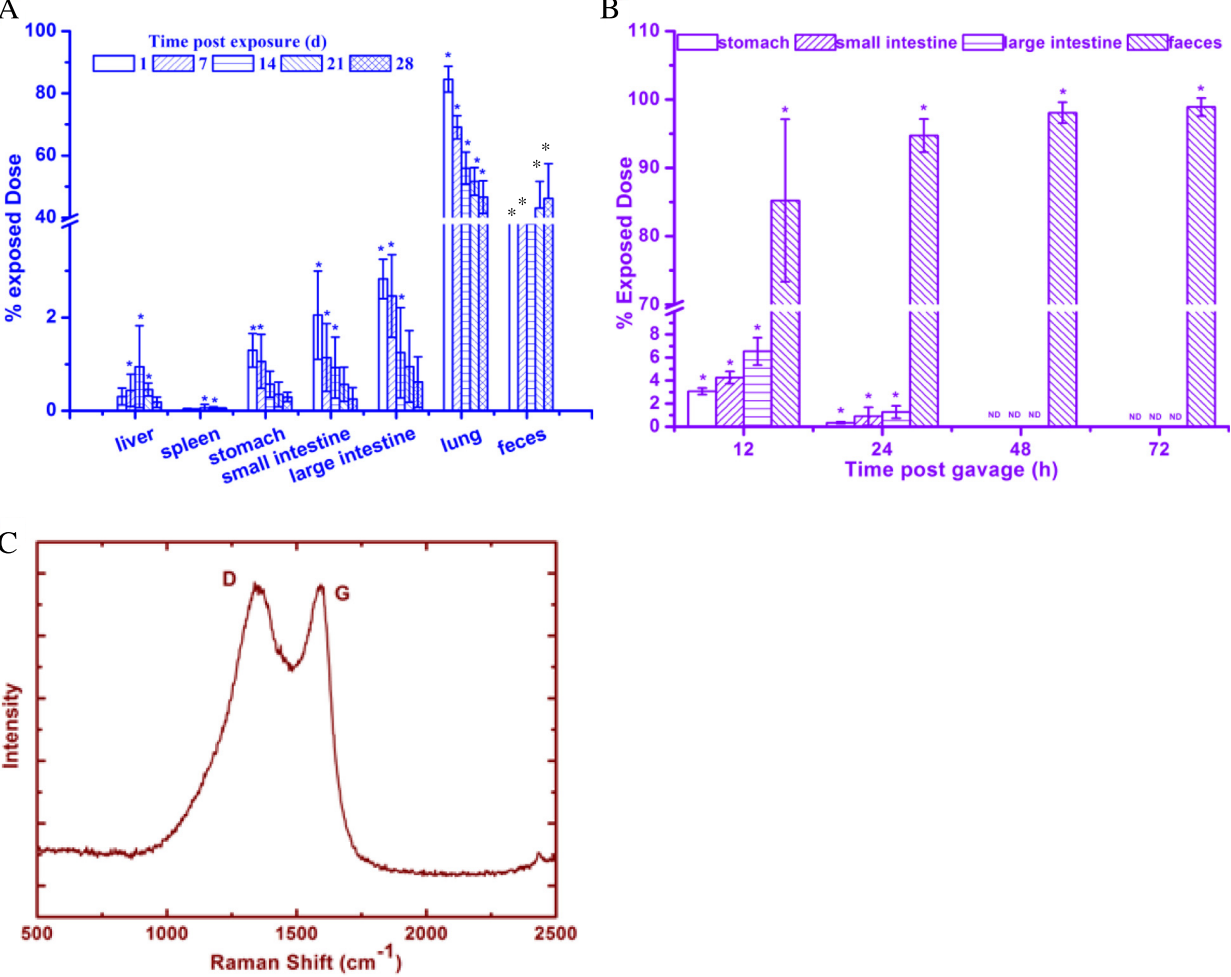
Biodistribution and clearance of ^14^C-graphene in male ICR mice after **a** intratracheally instillation or **b** oral gavage (5 μg ^14^C-graphene exposed) at different time points. **c** Raman spectra of the FLG obtained from stomach, small intestine, large intestine and lung after exposure for 1 d. Mouse feces were collected by metabolism cages. Data for the brain, heart, kidney, testis, muscle and blood for parts A and B and also the liver and spleen for part B were not shown because the radioactivity was always below the detection limit. The recovery of graphene in each organism tissue was in the range of 89 to 93 %. The data in the figure was corrected by these recovery values. The symbol‘*’indicates values that differed significantly from the control group at *P* ≤ 0.05. Data are presented as mean ± standard deviation (*n* = 5)

Distribution results after inhalation exposure for 1 day reveal that the radioactivity in the lung, large intestine, small intestine, stomach and feces was 85, 3, 2, 1.5 and 4.6 % of the exposed dose at 1 day (Fig. 2a), respectively; uncertainty values for biodistribution results are presented in the figures. The radioactivity in lung, large intestine, small intestine and stomach gradually decreased over time, while the radioactivity in the feces increased over time and was detectable in liver and spleen after 7 days post exposure. However, 47 % of the exposed dose remained in the lung after 4 weeks. Radioactive FLG concentrations in the brain, heart, kidney, testis, muscle and blood were always below the detection limit. To confirm translocation of the FLG, the large intestine, small intestine and stomach after exposure for 1 d (measured to be 1.3, 2.1, and 2.8 %, respectively, of the initial intratracheally instilled dose) were collected, washed and then characterized using Raman spectroscopy (Fig. 2c). We observed D and G bands which are distinctive of graphitic materials (the D band represents the disorder present in sp^2^-hybridized carbon systems, while the G band represents the stretching of C-C bonds), thereby confirming the presence of the FLG in each tissue [20]. Experiments were conducted to assess the relationship between FLG spiked to tissues and the intensity of the Raman signals. As shown in Additional file 1: Figure S1, neither the peak area nor height were linearly correlated with the concentrations of FLG tested (2 to 8 % of the initial intratracheally instilled dose of ^14^C-FLG). Thus, Raman spectroscopy using the method described in this manuscript was suitable to confirm the presence of FLG but was not quantitative. The potential for the formation of degradation products in tissues (liver, stomach, small intestine, large intestine and lung) was assessed for organisms 14 d after exposure using GC-MS, HPLC, and LSC, as described in the Method section. Additional chemical peaks were not found in the extraction solution using either HPLC or GC-MS. The radioactivity readings in the extraction solution after liquid scintillation counting were not statistically different from the background value. As such, detectable FLG degradation was not observed in any tissue. Additional experiments were performed to determine the recovery of potential FLG degradation products from organism tissues. Because it was unclear which degradation byproducts could be formed by metabolic processes in the organisms, FLG degradation products were produced by the Fenton reaction [21] and used to test the recovery. Results showed that unmodified FLG in the tissue was not extractable using this procedure, but that the recovery of the degradation products (spiked to liver tissues at a radioactivity concentration that was 1 % of the initial intratracheally instilled dose of ^14^C-FLG) ranged from 73 to 93 % (see Additional file 1: Figure S2). It is possible that the FLG was modified in the tissues (i.e., oxygen-containing functional groups were introduced to the surface of FLG), but additional specific characterization measurements are needed to determine such changes to FLG as FLG are not extractable from the tissues. The decreasing concentration of FLG in the liver during the 28 d exposure period may be attributable to FLG being translocated to other tissues or degradation products in the liver that were quickly excreted and therefore not detectable through our analyses.

**Fig. 3:**
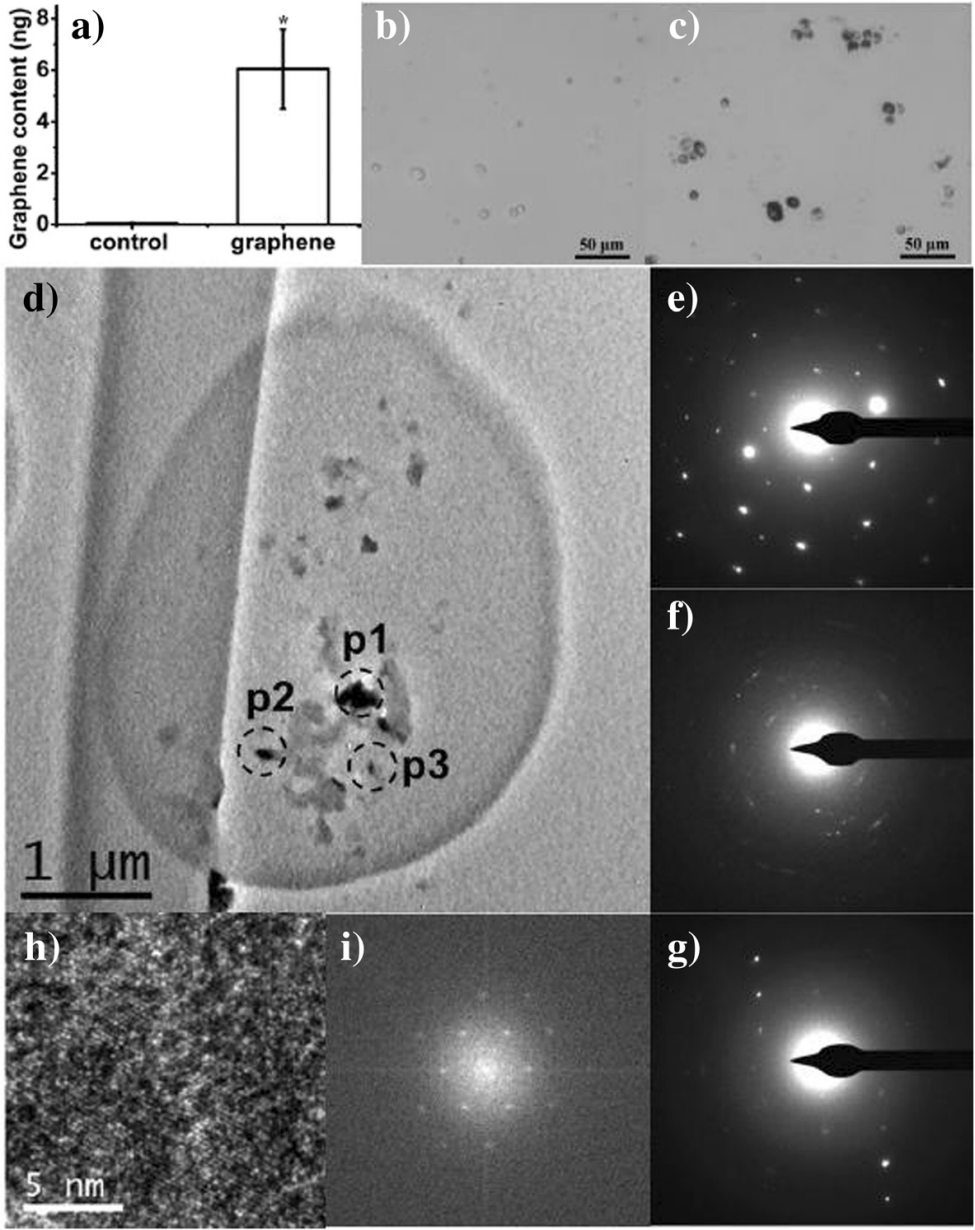
**a** FLG content in macrophages collected from the mice of the control experiment and FLG treated group (5 μg, 28 days), background radioactivity was subtracted; light microscope of macrophage collected from BALF of mice (**b**, control group; **c**, FLG-treated); **d**) TEM image of a whole BALF cell from FLG-treated mice; **e**), **f**) and **g**) SADPs taken from p1, p2 and p3 positions, respectively; **h**) High resolution TEM image taken from the p2 position; **i**) Fourier transfer spectra of Figure H. The symbol‘*’indicates values that differed significantly from the control group at *P* ≤ 0.05. Data are presented as mean ± standard deviation (*n* = 3)

The considerable FLG concentration measured in digestive organs (stomach, small intestine and large intestine), suggests that FLG was cleared from lung and delivered to other extrapulmonary organs. To explore this process further, bronchoalveolar lavage fluid (BALF) lavaged from graphene treated mice was collected and centrifuged. Cellular precipitate was washed using saline and resuspended for analysis using LSC. As shown in Fig. 3a, radioactivity was detected in the alveolar macrophages of the FLG treated mice, while the radioactivity of the cells in the control experiments that BALF was extracted from control mice (not exposed to FLG) was below the detection limit indicating the absence of detectable artifacts from the separation process. Fig. 3b and c provide visual evidence of FLG particles contained in the alveolar macrophages of exposure group but not the control group. The macrophages were sectioned and its electron micrograph was shown in Fig. 3d. Fig. 3d shows a low magnification transmission electron microscopy (TEM) image of macrophages containing black particles. These particles were confirmed to be FLG using selected area electron diffraction patterns (SADP), which showed well-defined diffraction spots confirming the crystalline structure of the particles (Fig. 3e, f and g), and high resolution TEM which revealed visible ordered graphite lattices and the interlayer distance is about 0.344 nm (Fig. 3h and i), which is the interlayer distance of graphite [22]. FLG was found in the cytoplasm of alveolar macrophages, which indicates FLG was phagocytized by the alveolar macrophages after intratracheal instillation. Thus, FLG could be cleared from lung through two possible routes: i) removal by mucociliary clearance and swallowed into the digestive system; ii) or elimination of the phagocytized FLG by alveolar macrophages through the tracheobronchial tree towards the larynx, a path previously shown to play an important role in eliminating nanoparticles from the lung [23, 24].

As shown in Fig. 2a, ~1 and 0.18 % of the exposed dose was also observed in liver and spleen at 14 days, suggesting that FLG administered to the lung has entered into blood circulation. FLG may pass through the air-blood barrier into blood and then be delivered to liver and spleen, or enter into the blood via adsorption through the gastrointestinal tract. To assess the possibility of gastrointestinal adsorption and to test FLG biodistribution after oral gavage, we gave mice a single dose of graphene via gavage and quantified the radioactivity in blood and major tissues during a 3 d exposure period (Fig. 2b). While the stomach, small intestine, large intestine, and feces contained 3, 4, 6 and 85 % of the exposed dose at 12 h, respectively, most of the FLG (>98 %) were excreted to the feces after 48 h exposure. No radioactivity was detected in blood, heart, liver, spleen, kidney, brain, lung, urine or testes after a single gavage, thus indicating that FLG are unable to be absorbed into blood circulation via the gastrointestinal tract at detectable concentrations. Similarly, Yang et al. reported that GO was not able to be adsorbed by the digestion system after oral feeding and became undetectable in all organs after 1 week; however, this studied using an external radioactive label which is a less reliable analytical approach [25]. Similarly, multiwall carbon nanotubes administrated by oral gavage were completely excreted after 12 h via feces, and there was no accumulation of nanoparticles in liver and spleen [26]. If a small fraction of the intratracheally instilled FLG was accidentally swallowed into the GI tract during administration of the suspension, this would only impact the 1 d results given the near complete excretion of orally administered FLG within 48 h. Thus, FLG found in the liver and spleen after intratracheal installation passed through the air-blood barrier and was then directly translocation into blood circulation [27–29]. Conhaim and coworkers reported that the lung epithelial barrier was best fitted by a three-pore–sized model, including a small number (2 %) of large-sized pores (400 nm pore radius), an intermediate number (30 %) of medium-sized pores (40 nm pore radius), and a very large number (68 %) of small-sized pores (1.3 nm pore radius) [30]. Considering the wide distribution range of graphene sizes in our study (see Fig. 1), a fraction of graphene likely passed the air-blood barrier and was translocated to liver and spleen. Li et al applied ^125^I labeled GO to study its distribution in Kunming mice by intratracheal instillation and found that the radioactivity was detected in blood and organs, including liver, spleen and thyroid gland [13]. However, this study only assessed the biodistribution after 12 h.

### Pulmonary toxicity of graphene

**Fig. 4:**
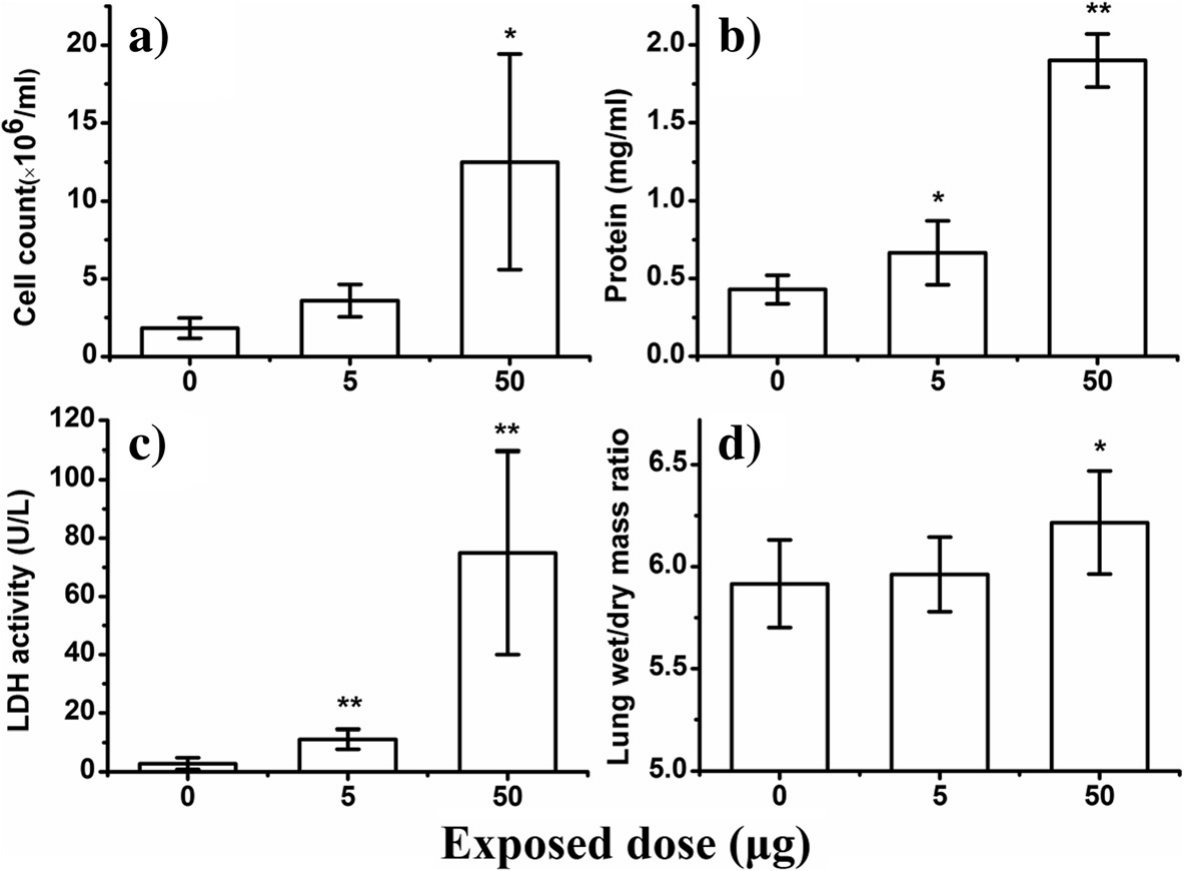
FLG causes acute pulmonary toxicity characterized mainly by cell injury and lung edema 24 h post exposure. **a** BALF total cell count; **b** BALF total protein; **c** LDH activity; **d** The lung wet/dry weight ratio used to evaluate the severity of lung edema. The symbol‘*’and‘**’indicates values that differed significantly from the control group at *P* ≤ 0.05 and *P* ≤ 0.01, respectively. Data are presented as mean ± standard deviation (*n* = 6)

We performed assays of neutrophil infiltration, cell injury, and lung edema to evaluate the potential acute pulmonary effects from different FLG doses (5 and 50 μg) (see Fig. 4) after 24 h post exposure. The total cell counts were five times higher in the tested group (50 μg) compared to the control group, which indicates that inflammatory cell infiltration likely occurred [31]. The observed difference of the BALF total protein and lactate dehydrogenase (LDH) level between the control group and the tested group of 50 μg was a factor of 4 and 27 fold higher, respectively, suggesting that the FLG had caused a degree of cell injury [32]. Because some studies show that nanoparticles may interfere with toxicity assays leading to artefacts [33, 34], additional experiments were conducted which confirmed that our results were not impacted by artefacts as discussed in the Additional file 1: Figure S3.

**Fig. 5:**
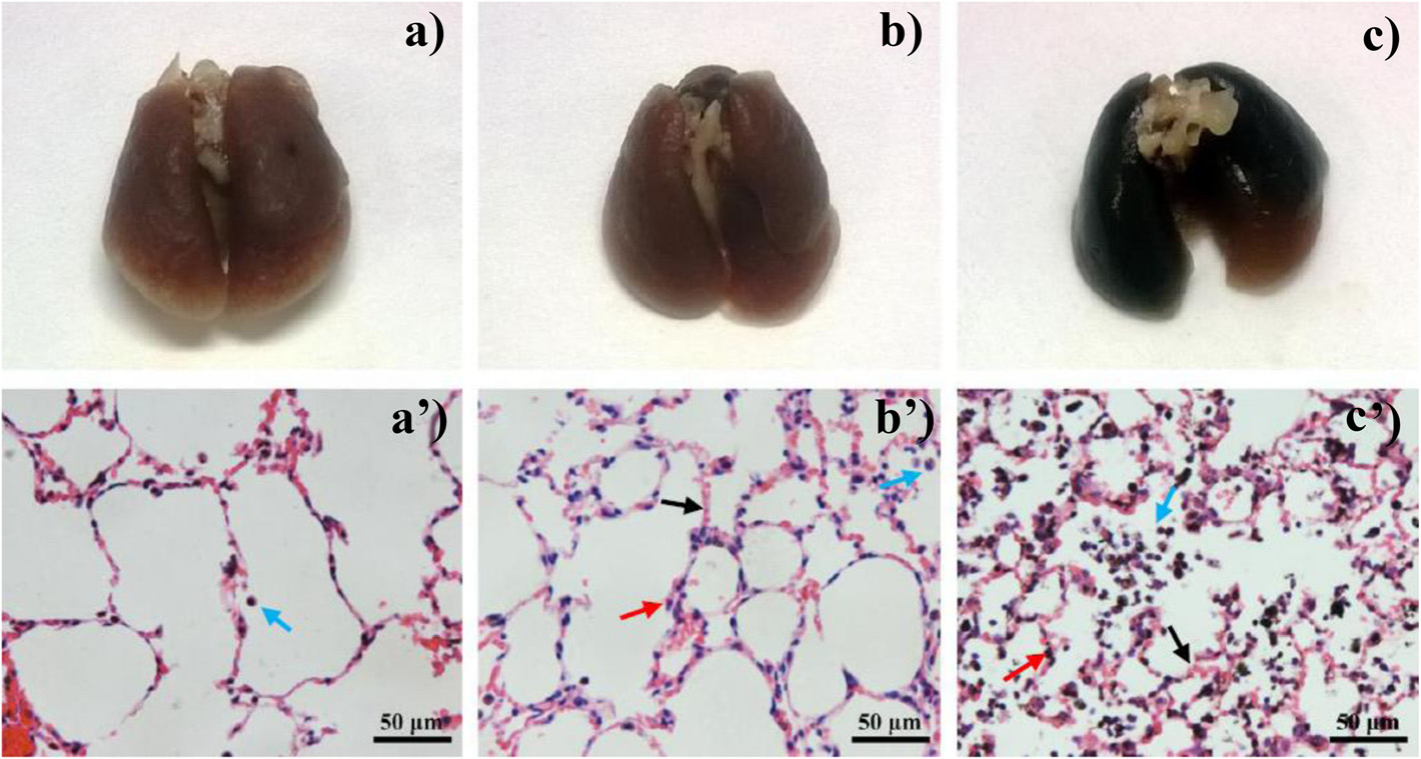
The morphological observation and representative H&E stained images of the lungs harvested 24 h post exposure (**a** and **a’** control group; **b** and **b’**, 5 μg exposed dose; **c** and **c’**, 50 μg exposed dose). Sections were analyzed blindly and representative images selected from 6 mice per treatment group are shown. Blue arrows: cells in alveoli; red arrows: parenchymal; black arrows: interstitial edema

The lung wet/dry mass ratio is a simple but useful indicator to assess severity of lung edema, which often arises from the leakage of fluid from capillaries into the interstitial and alveolar spaces and the loss of lung’s ability to pump fluid out of the space [35]. We found that exposure of 50 μg FLG caused a moderate pulmonary edema to mice as evidenced by the change in the dry to wet mass ratio (Fig. 4d). Lungs collected from mice of control and the tested dose groups (5 and 50 μg) were also analyzed by morphological and pathological observation (Fig. 5). The lungs treated with 5 μg FLG exhibit no abnormal appearance via morphological observation. However, most of the lung lobes (treated with 50 μg) turned black due to distribution of FLG throughout the lung (Fig. 5). Pathological observation of hematoxylin-eosin (H&E) stained lung sections suggested that the alveoli’s structure in control group was integrated and only few cells were found. However, lungs from FLG treated mouse exhibited mild to moderate interstitial edema and parenchymal edema, which was also evidenced by the change of lung wet/dry ratio (in Fig. 4d). This finding is supported by the presence of multiple lung macrophages in the alveolis of the high dose exposure group (Fig. 5). Similar results were reported in a study that examined the appearance and pathological section of lungs collected from mice which were treated with 50 μg dispersed graphene and found graphene uniformly distributed in the lung and only minimal lung inflammation [8]. However, Schinwald et al found graphene induced ganulomatous lesion formation and the exposure dose in their study was also 50 μg [6].

**Fig. 6:**
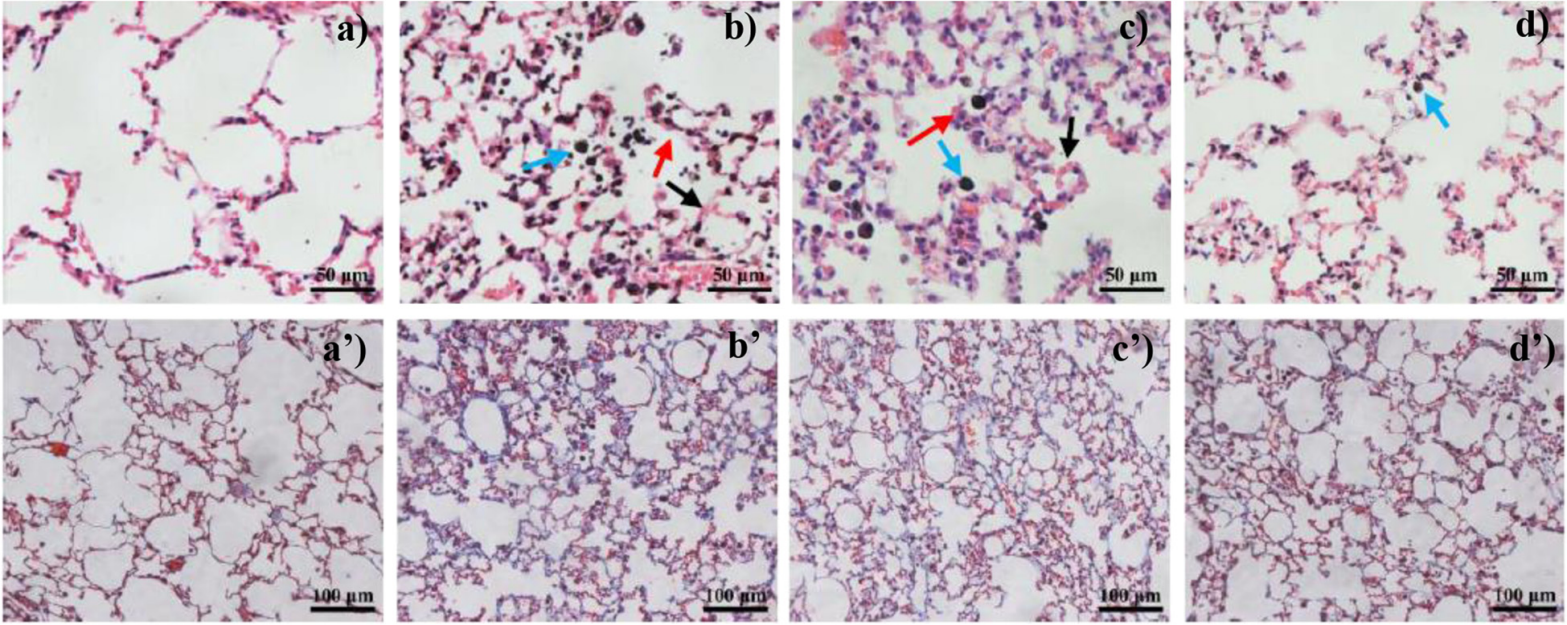
H&E (**a**, **b**, **c**, **d**) and Masson (**a’**, **b’**, **c’**, **d’**) stained lung sections of mice which were exposed 50 μg FLG at different time points post exposure (**a** and **a’** were control group at 1 day post exposure; **b** and **b’** were 1 day post exposure; **c** and **c’** were 7 days post exposure; **d** and **d’** were 28 days post exposure). Sections were analyzed blindly and representative images selected from 6 mice per treatment group are shown. Blue arrows: cells in alveoli; red arrows: parenchymal; black arrows: interstitial edema

To explore the time-dependent toxicity of graphene after intratracheal instillation, H&E and Masson staining were applied to examine pathological changes of lung tissue. As shown when control Fig. 6 (a) is compared to Fig. 6 (b), moderate interstitial and parenchymal edema was observed in H&E stained lung sections after exposure for 1 day. Severe inflammatory cell infiltration were also observed, which was characterized by substantial quantities of cells in the pulmonary alveoli. Though the severity was reduced, minimal pulmonary edema and inflammatory infiltration was also observed after 7 days (Fig. 6 (c) compared to control Fig. 6 (a)). However, no obvious abnormal pathological changes and lung structure damage were found in lung sections 28 days later despite the continued presence of approximately 47 % of the initial FLG dose in the lungs which are observed as small black areas in Fig. 6 (d). Similar results were previously reported for graphene platelets which only caused minimal inflammation in mouse lungs after 6 weeks exposure [9].

We further examined Masson stained lung sections for the evidence of fibrosis at 1, 7 and 28 d. However, there was little evidence of lung fibrosis in mice treated with FLG, regardless of different exposure time (Fig. 6). Aggregates of carbon nanotubes and aggregated graphene have been shown to induce peribronchiolar lung fibrosis 21 d or longer after their administration [8, 36]. It is possible that improving the dispersion state of graphene such as the usage of Tween 80 to disperse FLG in this study may reduce the likelihood of lung fibrosis formation. Importantly, it was found that graphene dispersed with a pluronic surfactant did not show fibrogenic effects in cells or mice while these effects were observed when the same nanomaterial was suspended with bovine serum albumin [37]. Previous studies have shown that GO oxide can cause damage to macrophages, and that the lateral size of the graphene can impact the toxicity to cells and mice with larger GO particles having higher toxicity [37–40]. Additional work is needed to compare FLG to GO of an individual sheet but with similar lateral size and surface chemistry (e.g., oxygen content) to assess the impact of GO thickness on the toxicity response.

### Influence of graphene on intestinal flora

**Fig. 7:**
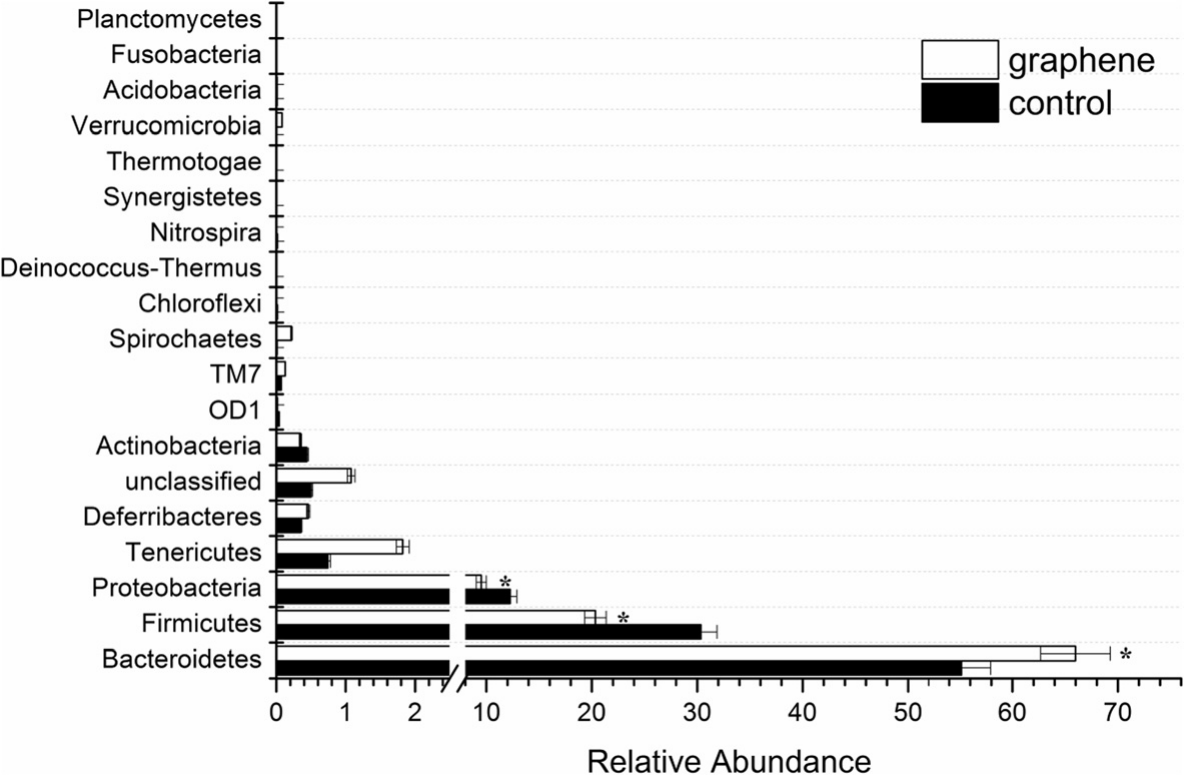
Relative abundance of mice intestinal microbial community structure at phylum level. The symbol‘*’indicates values that differed significantly from the control group at *P* ≤ 0.05. Data are presented as mean ± one standard deviation (*n* = 3)

FLG was excreted via the intestinal tract regardless of exposure via oral gavage or intratracheally instillation (see Fig. 2). During passage of FLG through the gut tract, graphene may impact the intestinal microbial community structure. Bacteroidetes, Firmicutes and Proteobacteria are the three main microbial communities in gut of mice and account for more than ninety percent of the total gut flora. While the quantity of Proteobacteria was similar between the control and graphene-treated mice (Fig. 7), the relative abundance of the other two predominant bacterial communities in mice statistically differed. The relative abundance of Firmicutes in mice exposed to FLG was decreased by 10 ± 1.2 % compared to the control group while the relative abundance of Bacteroidetes was increased (see Fig. 7). Previous studies revealed that obesity is associated with changes in the relative abundance of the two dominant bacterial divisions, Bacteroidetes and Firmicutes [41]. However, we did not find that the FLG exposure affected the body weight of mice by monitoring the body weight change of mice during exposed period (Additional file 1: Figure S4), potentially as a result of an exposure period too short to observe the weight change or a lack of sensitivity for mice to this degree of change in the microbiome. A previous study demonstrated that carbon nanotubes have broad-spectrum antibacterial effects against gut bacteria [42], but this is the first *in vivo* report of FLG’s biological effects on gut bacteria. It is important to note that previous studies have shown toxicological effects of graphene oxide on bacteria [43, 44]. While this study focused on the intestinal microbial community structure through analysis of the DNA in the feces, it would be important to also investigate potential toxicological effects on the remaining bacteria in the gut tract. In contrast to studies testing the direct toxic effects of graphene family materials on bacteria in *in vitro* systems, graphene during passage through the gut tract of organisms after intratracheal installation may be covered by surfactants or biomolecules from the lung or digestive system and thus have a different toxicity. Further research is also needed to clarify the extent to which long-time graphene exposure in the digestive system would alter the intestinal microbial community structure, cause body weight changes, and potentially have a harmful effect on the test organisms.

## Conclusions

Intratracheally instilled graphene was mainly retained in the lung with 47 % remaining after 4 weeks. Exposure to non-labeled graphene resulted in dose-dependent acute lung injury and pulmonary edema, but these effects were alleviated with time despite the continued presence of graphene in the lungs. Intratracheally instilled graphene was redistributed to the liver and spleen by passing through the air-blood barrier, a finding supported by the results of oral gavage experiments which did not show detectable absorption through the gastrointestinal tract. The *in vivo* distribution, elimination, and toxicity results provided here measured using a robust quantitative method support the human health risk assessment of graphene.

## Methods

### Materials and animals

Synthesis, purification, and characterization of ^14^C-labeled FLG were described in our previous studies [17, 21, 45]. Briefly, graphene sheets were successfully synthesized by graphitization and exfoliation of sandwich-like FePO_4_/dodecylamine hybrid nanosheets and then purified using hydrochloric acid to remove the iron catalysts to below the limit of detection using ICP-OES (>5 μg/L). Liquid scintillation counting (LSC) and mass spectrometry analyses could not detect the formation of carbon-14 byproducts from the synthesis, purification, or dispersion processes [17].

^14^C-labeled FLG powder was dispersed in 0.1 % Tween 80 saline and ultrasonicated in an ice-water bath for 3 h to 3.5 h (100 W, JY88-II, Nanjing Immanuel Instrument Equipment Co.). Probe sonication was performed using a 3 s “on”/ 2 s “off” pulse sequence with a probe tip that placed approximately 0.4 cm from the bottom of the container [21]. Our preliminary results of XPS confirmed that sonication using this procedure for up to 10 h did not change the elemental composition of the FLG (see Additional file 1: Figure S5). Prior to administration, graphene solutions were re-dispersed using ultrasonication for 30 min. Light microscopy, DLS and AFM was applied to characterize the dispersion state and particle size distribution of suspended FLG in the dispersion medium. For optical microscopy, a droplet of graphene suspension (0.5 mg/mL) was placed on a glass slide and covered with a coverslip. Images were captured at 40× magnification using NIS-Elements software (Nikon, Japan). The particle size distribution of dispersed FLG was characterized by using DLS (ZetaPALS, Brookhaven Instruments Corp., USA) at the scattering angle θ = 90°. The standard spherical particle models were used to convert the DLS autocorrelation functions to z-average sizes. Five runs and one minute run duration were set for each measurement. AFM images of FLG were recorded using a MultiMode V8 scanning probe microscope (Bruker, German). Samples for AFM were prepared by dropping the highly dispersed aqueous suspension of FLG on a freshly cleaved mica surface. After the samples were dried, the AFM images were measured using a ScanAsyst Mode. The commercially available AFM cantilever tips with a force constant of ∼ 0.4 newton/m and resonance vibration frequency of ∼ 70 kHz (Bruker, USA) were used.

Four-week-old male ICR mice were purchased from the laboratory animal research center of Jiangsu University. The animals were housed in plastic cages, and provided with water and feed ad libitum. Before being employed in experiment, all animals were acclimated to the laboratory environment for 1 week. Ambient conditions were at 25(±3) °C, 50(±5)% relative humidity, and a 12/12 h light/dark cycle. The mice were treated humanely according to Regulations on Laboratory Animals (China), and the protocols were approved by the Animal Care and Use Committee of NGH.

### Distribution of graphene in mice via intratracheal instillation

Twenty-five mice were randomly divided into five groups of five mice each. Mice were anesthetized with 10 % chloral hydrate (280 to 350 mg/kg body weight). Fifty μL of ^14^C labeled FLG suspension (0.1 mg/mL, 1.3 × 10^5^ dpm) was delivered directly into the lung of each mouse via intratracheal instillation. The dose of FLG in the lung was 5 μg. After exposure, each mouse was housed individually in a metabolism cage to collect feces separately. One group of mice was sacrificed at 1, 7, 14, 21 and 28 days post exposure by dislocation of the vertebrae. The lungs of mice were then lavaged three times with 800 μL of 4 °C sterile saline to collect BALF [46]. Then, their tissues, including brain, heart, lung, liver, spleen, kidney, testis, small intestine, large intestine, muscle and blood were harvested. All tissues, blood and feces were freeze-dried (Labconco, America) and ground into powder. 25 mg to 35 mg of ground tissue, blood, or feces was combusted using a biological oxidizer (BO, OX-500, Zinsser Analytic, Germany) at 900 °C for 4 min under a stream of oxygen gas running at 360 mL/min. The ^14^CO_2_ released during the combustion process was captured in alkaline carbon-14 scintillation cocktail (Zinsser Analytic, Germany) and then analyzed by LSC. The recovery of graphene in each organism tissue was in the range of 89 to 93 %; the reported data for each tissue was corrected by these recovery values. The minimum detection limit of LSC and BO was measured to be 3.85 ng ^14^C graphene and 0.14 ng ^14^C graphene per milligram tissue, respectively, values which determined from the signal from blank samples plus three times the standard deviation of the blank samples.

The FLG in the stomach, small intestine, large intestine and lung at 1d was respectively collected, washed in sequence using DI water, dichloromethane, n-hexane and dichloromethane, and then characterized using Raman spectroscopy (XploRA PLUS system, Horiba Scientific, 532 nm incident radiation). Different doses of ^14^C-FLG were spiked with 4 mg liver (dry mass) to test the Raman response of FLG (see Additional file 1).

To assess potential metabolic products from the FLG, each tissue (liver, stomach, small intestine, large intestine and lung) at 14 d after exposure was freeze-dried, ground into powder, and extracted in sequence using dichloromethane (5 mL), n-hexane (5 mL), and dichloromethane (5 mL). These solutions were recombined, and subjected to anhydrous sodium sulfate to remove the water. Then, the sample was dried using a gentle nitrogen stream and reconstituted in methanol and dichloromethane (4:1, v/v) for HPLC and GC-MS analysis, (see Additional file 1). The radioactivity in the extraction was also analyzed using LSC. To test the recovery of this extraction procedure, a known amount (1 % of the initial intratracheally instilled dose) of ^14^C-degradations products produced by the Fenton reaction [21] or un-modified FLG were spiked to the powder of livers from control mice and the extraction procedure was performed (see Additional file 1).

Low speed centrifugation (850 g, 10 min) was performed to obtain cellular fraction of BALF. The obtained cellular precipitate was washed >5 times using saline until the radioactivity in the eluate was not detectable (<100 dpm) by LSC. Then, the cellular precipitate was resuspended in saline and sonicated by ultrasonic processor to break up cells. The FLG was quantified by measuring the radioactivity in the cell suspension using LSC. To assess possible adsorption of FLG on cell membranes during the process to separate the FLG from the BALF cells or incomplete FLG removal, control experiments were conducted. BALF extracted from control mice (not exposed to FLG) were mixed with 50 μL ^14^C labeled FLG suspension. Within five minutes after mixing, low speed centrifugation was performed to obtain cellular fraction, the BALF cellular precipitate was treated by following the procedures mentioned above and the FLG was quantified. A drop of cell suspension was placed on a glass slide and covered with a cover glass. Then, a glass slide was put under an optical microscopy and bright field images of BALF cells were taken. Meanwhile, parts of the obtained BALF cells were washed with physiological saline, prefixed in 3 % glutaraldehyde at 4 °C overnight, and then post-fixed in 1 % osmium tetroxide. After dehydration and resin embedding, BALF cells were sectioned to 50 to 60 nm thick, and stained with uranyl acetate and lead citrate [47]. The structure of graphene particles in cells was identified using high resolution TEM imaging together with corresponding SADP on a 200 kV FEI Tecnai TF20 FEG-TEM [48].

### Distribution of graphene in mice via oral gavage

Twenty mice were randomly selected and 100 μL ^14^C labeled graphene suspension (0.1 mg/mL, 2.6 × 10^5^ dpm) was delivered into the stomach of each mouse via oral gavage. The dose of FLG was 10 μg in total. After one single exposure, each mouse was housed individually in a metabolism cage to collect feces and urine separately. At each sampling time (12, 24, 48, 72 h), 5 mice were sacrificed and their blood and major tissues, including brain, heart, lung, liver, spleen, kidney, testis, small intestine, large intestine and stomach were harvested. All organ samples and blood were freeze-dried (Labconco, America) and ground into powder. Twenty-five mg to 35 mg powder of tissues and feces and 0.1 mL urine was combusted using BO and the released ^14^CO_2_ analyzed by LSC. The recovery of graphene in urine and blood was 97(±1.2)% (*n* = 3); uncertainties always indicate one standard deviation.

### Pulmonary toxicity of graphene

Acute toxicity experiments of FLG to lung were performed to assess potential effects at two graphene dosages. Eighteen mice were randomly assigned to control and two FLG-treated groups. After being anesthetized, mice were intratracheally instilled with 50 μL of a control solution without graphene or a non-labeled FLG suspension (0.1, or 1 mg/mL) that were prepared as described above for the carbon-14 labeled FLG. Twenty-four h later, all mice were killed by dislocation of vertebrae. BALF was collected as described above and then centrifuged for 10 min (210 g, 4 °C) to obtain the cellular fraction and supernatant. The cellular fraction was resuspended in 1 % BSA/saline. Twenty μL of the cell suspensions were pipetted onto a hemocytometer chamber, and the cells were counted manually. The supernatant was kept at −80 °C for total protein and LDH assays using commercial kits (Nanjing Jiancheng Bioeng. Inst., China). To calculate the lung wet/dry mass ratio, the left lung was cut and rinsed using saline. Water on the surface of lung was drained with filter paper. The lung was weighed, then dried in a vacuum freeze drier (Labconco, America) for 72 h and reweighed to determine wet/dry mass ratio. Histological examination was also performed to evaluate toxicity of graphene to the lung. The right lung was removed and fixed in 10 % neutral buffered formalin overnight. The tissue was embedded in paraffin, sectioned and stained with H&E to show gross pathology. Images at 40× magnification were taken using NIS-Elements software (Nikon, Japan) to show higher magnification areas of the lung sections.

Eighteen additional mice were intratracheally instilled with 50 μL of a 0.1 mg/mL FLG suspensions for longer-term toxicity testing. Lungs of mice were harvested and preserved in formalin for histological examination after 1, 7 and 28 days post exposure. Six mice were sacrificed at each sampling time. Another 6 mice were exposed to 50 μL 0.1 % Tween 80 saline as control. H&E together with Masson staining method was used to examine possible pathological changes and lung fibrosis of graphene exposed mice [49].

### Impact of graphene on intestinal flora

Eighteen mice were divided into two groups. Nine mice of one group were gavaged daily with 0.4 mL of 0.1 % Tween 80 saline as control group, while the other group were gavaged with 0.4 mL of 2.5 μg/mL FLG suspensions (suspended in 0.1 % Tween 80 saline as described above). After gavage for 28 days, mice were fasting for 12 h before dissection. Nine mice of each group were randomly divided to be three sets and each set of parallel has three mice. The germ free feces of each set were collected from their rectums, combined, and stored in−20 °C. One hundred mg feces samples from each set were measured and three 100 mg samples were obtained. Total genomic DNA was extracted from each of the three samples using FastDNA Soil Kit (MP Biomedicals, USA) following the manufacturer protocol. The concentration and quality of the extracted DNA were determined using spectrophotometry (NanoDrop, USA) to ensure that the DNA concentration in the samples was greater than 200 ng/μg for the following experiments. The samples with >200 ng/μg DNA were selected and amplified by Polymerase Chain Reaction. The amplified DNA samples were purified using DNA fragment purification kit ver 4.0 (Takara, Japan) according to the recommended protocol. About 10 μg (quantified by Qbit) of purified DNA sample was sent to Jiangsu Zhongyijinda Analytical & Testing CO., LTD. (Yixing, China) for high-throughput sequencing using Illumina’s Miseq platform. Generated 16S rRNA gene sequences were processed using Mothur software.

### Statistical analysis

All statistical analyses were performed using SPSS 18.0 (PASW Statistics, IBM Company); differences were considered statistically significant at *p* < 0.05. Errors always represent one standard deviation and the numbers of samples are reported with the standard deviation.

## Additional file

Additional file 1:
**Additional description of certain experimental procedures.** **Table S1.** **Figure S1.** Raman spectra of liver tissue mixed with different FLG masses. **Figure S2.** HPLC chromatogram of products resulting from FLG reacted with Fenton reagent and the possible structures of the products. A list of publications on the biological effects of graphene and functionalized graphene. **Figure S3.** Assessment of graphene interference with the protein and LDH assay. **Figure S4.** Body weight of mice in control and graphene-treated groups during exposure period. **Figure S5.** XPS spectrum of the FLG at different sonication time. (DOC 483 kb)

## Abbreviations

GO: graphene oxide
FLG: few layer graphene
LSC: liquid scintillation counter
DLS: dynamic light scattering
AFM: atomic force microscopic
TEM: transmission electron microscopy
BALF: bronchoalveolar lavage fluid
BO: biological oxidizer
LDH: lactate dehydrogenase
H&E: hematoxylin-eosin
SADP: selected area electron diffraction patterns
