# Supplementary material for: Biodistribution and toxicity of radio-labeled few layer graphene in mice after intratracheal instillation
Source: Part Fibre Toxicol. 2016 Feb 11;13:7. doi: 10.1186/s12989-016-0120-1 (PMC4750184; doi:10.1186/s12989-016-0120-1)
Supplement: Additional file 1: — Additional description of certain experimental procedures. Table S1. Figure S1. Raman spectra of liver tissue mixed with different FLG masses. Figure S2. HPLC chromatogram of products resulting from FLG reacted with Fenton reagent and the possible structures of the products. A list of publications on the biological effects of graphene and functionalized graphene. Figure S3. Assessment of graphene interference with the protein and LDH assay. Figure S4. Body weight of mice in control and graphene-treated groups during exposure period. Figure S5. XPS spectrum of the FLG at different sonication time. (DOC 483 kb) [file 12989_2016_120_MOESM1_ESM.doc]

**Supplemental Material**

**Biodistribution and Toxicity of Radio-labeled Few Layer Graphene in Mice after Intratracheal Instillation**

Liang Mao^*^, Maojie Hu, Bingcai Pan^*^, Yongchao Xie, Elijah J. Petersen

* E-mail: [lmao@nju.edu.cn](mailto:lmao@nju.edu.cn) or [bcpan@nju.edu.cn](mailto:bcpan@nju.edu.cn) .

**Table of Contents**

**I. Additional description of certain experimental procedures………………….....Page 3**

**II. A list of publications on the biological effects of graphene……………………..Page 6**

Table S1

**III. Additional figures…….…………………………………………………………..Page 7**

Figure S1through S5.

**I. Additional description of certain experimental procedures**

**HPLC analysis.** An Agilent 1200 Series High Performance Liquid Chromatograph (HPLC) equipped with VWD and fluorescence detectors was used to analyze the samples. Separation was performed on an Agilent C18 reverse phase column (250 × 4 mm, 5 μm, Ecllpse XDB- C18, Agilent, USA). The mobile phase was acetonitrile (70%) and water (30%), and eluted at 1 mL/min. Injection volume was 20 μL, and the detection wavelength for VWD detector was 230 nm. The excitation and emission wavelengths were 222 nm and 305 nm, respectively, for the fluorescent detector.

**GC-MS analysis.** GC/MS characterization was performed on a Thermo Finnigan Trace gas chromatograph equipped with a Polaris Q ion trap tandem mass spectrometer (GC-ITMS/MS, Thermo, Finnigan, USA). A DB-5 fused-silica capillary column (30 m × 0.32 mm i.d., 0.25 μm film thickness) was used for GC separation, and helium was used as the carrier gas at 1 mL/•min. The oven temperature was initially 60°C for 1 min, increased to 150°C at 10°C/min, to 260°C at 20°C/min, to 320°C at 5 °C/min and finally held at 320°C for 8 min. The injector, interface, and source temperatures were set at 280°C, 280°C, and 230°C, respectively.

**Raman spectrum after addition of different FLG masses.** A certain volume of the FLG stock solution was measured to achieve the desired doses of FLG (100, 200 and 400 ng) and mixed with 4 mg of liver (dry mass). The mixture was ground into a powder and then analyzed using Raman spectroscopy.

**Recovery of the degradation products from the FLG.** Samples at initial ^14^C-FLG concentrations of 500 μg L^-1^ were prepared to characterize the reaction products of FLG via the Fenton reaction as described in our earlier study [1]. The reactions were performed in 40-mL glass vials equipped with polytetrafluoroethylene (PTFE)-lined screw caps and incubated on a rotary shaker operating at 150 rpm at 25 °C. Each reactor contained a 20 mL reaction medium that was adjusted to pH 4. Fifty- μmol L^-1^ Fe^3+^ and 200 mmol L^-1^ H_2_O_2_ were added to each reactor to initiate the reaction. After incubation for 3 days, 10 mL of the aqueous phase from the reaction mixture was sampled and sequentially extracted three times using 10 mL dichloromethane each time. The extracted solutions were combined, dried by rotary evaporation, and then the residue was dissolved in methanol. The reconstituted samples were completely transferred into 1.8-mL vials and then each was reduced to 0.5 mL using a gentle stream of nitrogen gas.

A certain volume of the above solution was mixed with the homogenate of liver (1.4 g dry mass of liver) to yield a total radioactivity that was 1% of the initial intratracheally instilled dose of ^14^C-FLG. Then, the mixture was freeze-dried, ground into powder, and extracted in sequence using dichloromethane (5 mL), n-hexane (5 mL), and dichloromethane (5 mL). These solutions were recombined, and subjected to anhydrous sodium sulfate to remove the water. Then, the sample was dried using a gentle nitrogen stream and reconstituted in methanol for HPLC and LC-MS/MS analysis. Experiments were performed in triplicates.

**Interference of graphene on protein and LDH assay.** Additional control experiments were performed to examine the possibility of graphene interference in the protein and LDH measurements. Before testing graphene interference in the protein and LDH assays, the largest possible graphene concentration in the BALF collected from graphene exposed mice was calculated. According to the distribution results in Figure 2A of the text, ~90.8% of exposed graphene was detected in tissues 1 day post exposure. Thus, a conservative estimate assuming all of the unaccounted for radioactivity was in the BALF would indicate that ~9.2% of the exposed graphene was in the BALF since lungs were lavaged before the radioactivity of lung were measured. This would lead to maximum graphene concentrations of 0.192 μg/mL and 1.92 μg/mL (the volume of BALF is 2.4 mL) for the mice exposed to 5 μg and 50 μg graphene exposed mice, respectively. For the protein content in BALF measurements, the estimated maximum concentration of graphene in the reaction system was 0.0073 μg/L and 0.073 μg/L for 5 μg and 50 μg graphene exposed mice, respectively. Similarly, the maximum estimated graphene concentration is 0.011 g/L and 0.11 g/L of 5 μg and 50 μg graphene exposed mice for the LDH assay, respectively.

Protein and LDH assays were performed with the same basic procedure. A standard curve was calculated for each 96-well microplate assay and microplates were only used if the standard curve had an R^2^ > 0.99. First, we determined if the presence of graphene would induce the production of fluorescence or color without addition of an analyte. Different concentrations of graphene were added to the kit reagents and the assay was run according to manufacturer’s instructions. The final concentrations of graphene tested in the reaction system were 0, 0.5, 1, 2 and 4 mg/L and 0, 0.3, 0.6, 1.2, 2.4 mg/L for the protein and LDH assays, respectively. Secondly, we simulated a more realistic assay situation by adding a known concentration of an analyte. 500 μg/mL BSA for the Bicinchoninic (BCA) assay or 200 U/L LDH for the LDH assay was added to the reaction system in addition to the graphene and kit reagents. Control experiments without added graphene were also conducted. The results showed that graphene would indeed affect protein and LDH assays if the graphene concentration in the test media was above 0.3 mg/L (Figure S1 of SI), a concentration greater than the maximum that the BALF cells would be exposed to in this study. Therefore, the potential bias from graphene interference would not alter the conclusions of the results from the protein and LDH assays of BALF in this study.

**Supplemental Material, Table S1.** A list of publications on the biological effects of graphene and functionalized graphene

| Material | dose & route | distribution | biological effects | Ref |
| --- | --- | --- | --- | --- |
| graphene,  1-10 layer | 50 μg/mouse  （PA.） | N | Inflammatory response and granulomatous lesions at 1 day; continuous inflammatory response at 1 week. | a |
| graphene, N | 10 mg/L  （I.） | N | Inflammatory response; microgranulomas. | b |
| GO,1-6 layers  (0.5-2.0 nm) | 50 μg/mouse  (II.) | N | Severe lung inflammation at 1 d and persistent at 21 d; little evidence of lung fibrosis. | c |
| graphene, 4-15 layers (1.2-5.0 nm) | 50 μg/mouse  (II.) | N | Minimal lung inflammation at 1 day; peribronchial inflammation and mild fibrosis after 21 days. | c |
| graphene, 1-10 layers | 50 μg/mouse  (PA.) | N | Acute inflammation in mice lungs at 1 day; minimal inflammation in mouse lungs after 6 weeks. | d |
| GO,  ~3 layers | 10 mg/kg bw  （II.） | Y (12 hr) *^a^* | Time and dose dependent toxicity to lung; diffuse pulmonary fibrosis at 1 and 3 months. | e |
| graphene,  4 layers | 5 and 50 μg/mouse  (II.) | Y (28 day) *^a^* | Acute lung inflammation at 1 day; no obvious lung structure damage after 28 days | this  study |

Abbreviation: graphene (G); pharyngeal aspiration (PA.); inhalation (I.); intratracheally instillation (II.); body weight (bw); not available (N) and available (Y); references (Ref) in the text. Ref: a (Schinwald A et al. 2012); b (Ma-Hock L et al. 2013); c (Duch MC et al. 2011); d (9. Schinwald A et al. 2013); e (Li B et al. 2013).

*^a^* *In vivo* distribution and excretion of graphene in mice up to 12 h (or 28 d).

Figure S1. Raman spectra of liver tissue mixed with different FLG masses.

**C**

**H**

**2**

**O**

**H**

**H**

**O**

**H**

Figure S2. HPLC (XDB C18 column) chromatogram of products resulting from FLG reacted with Fenton reagent and the possible structures of the products. Detailed information on the products identification using HPLC and LC-MS/MS were previously reported [1]. The recovery of the three products was 73.3%±5.2%, 78.5%±3.3% and 93.3%±6.3% (mean ± standard deviation values; n=3).


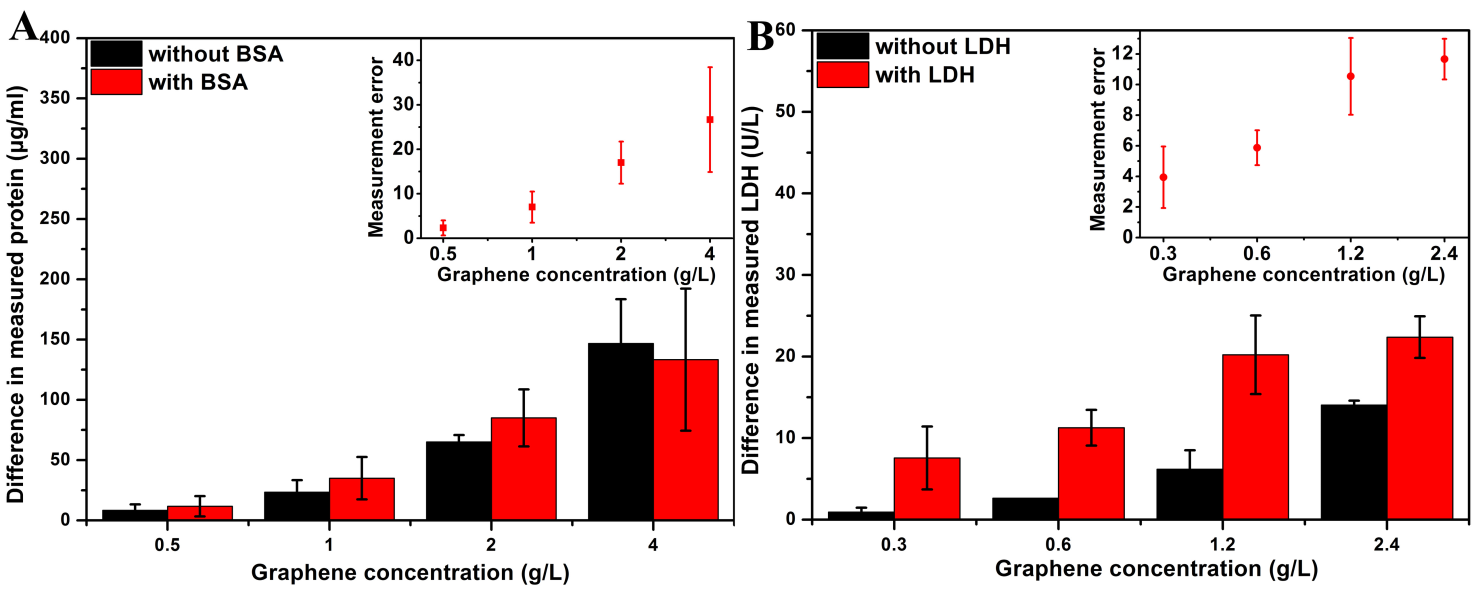


**Figure S3.** Assessment of graphene interference with the protein and LDH assay. (A) BCA (bicinchoninic) protein assay, the concentration of BSA added to reaction system was 0 μg/mL (without BSA) or 500 μg/mL (with BSA); (B) LDH assay, the activity of LDH added to reaction system was 0 U/L (without LDH) or 200 U/L (with LDH). The “difference in measured protein or LDH activity” parameter was calculated by subtracting the actual concentration of protein or LDH activity added to the reaction system from the value measured by the assay. The insert for part A illustrates the percentage of protein measurement error (difference in measured protein divided by 500 μg/ml BSA) caused by graphene interference when 500 μg/mL BSA was added into reaction system. A similar graph is shown in the insert for part B but for the addition of 200 U/L of LDH to the test system. Data are presented as mean ± one standard deviation (n=3).

**Figure S4.** Body weight of mice in control and graphene-treated groups during exposure period. Data are presented as mean ± one standard deviation (n = 9). t-tests were performed using SPSS 18.0 (PASW Statistics, IBM Company) and no significant difference was found between control and graphene treated mice.

Figure S5. XPS spectrum of the FLG at different sonication time. The right spectra was the relative content of oxygen after sonication for different times.

1. Feng YP, Lu K, Mao L, Guo XK, Gao SX, Petersen EJ. Degradation of C-14-labeled few layer graphene via Fenton reaction: Reaction rates, characterization of reaction products, and potential ecological effects. Water Res. 2015; 84: 49-57.
